# Micro-Ultrasound in the Diagnosis and Staging of Prostate and Bladder Cancer: A Comprehensive Review

**DOI:** 10.3390/medicina58111624

**Published:** 2022-11-10

**Authors:** Francesco Paolo Calace, Luigi Napolitano, Davide Arcaniolo, Marco Stizzo, Biagio Barone, Felice Crocetto, Michelangelo Olivetta, Ugo Amicuzi, Luigi Cirillo, Andrea Rubinacci, Arturo Lecce, Savio Domenico Pandolfo, Nunzio Alberto Langella, Francesco Persico, Francesco Trama, Carmelo Quattrone, Francesco Bottone, Lorenzo Spirito, Marco De Sio, Celeste Manfredi

**Affiliations:** 1 Unit of Urology, Department of Woman, Child and General and Specialized Surgery, University of Campania “Luigi Vanvitelli”, 80131 Naples, Italy; 2 Unit of Urology, Department of Neurosciences, Reproductive Sciences, and Odontostomatology, University of Naples “Federico II”, 80131 Naples, Italy; 3 Department of Urology, AORN Antonio Cardarelli, 80131 Naples, Italy; 4 Department of Surgical and Biomedical Science, Andrological and Urogynecological Clinic, Santa Maria Terni Hospital, University of Perugia, 05100 Terni, Italy

**Keywords:** micro-ultrasound, high resolution, prostate, bladder, cancer, tumor

## Abstract

*Background and Objectives*: Multiparametric magnetic resonance imaging (mpMRI) of the prostate and prostate-specific membrane antigen positron emission tomography (PSMA PET) are some examples of how the advancement of imaging techniques have revolutionized the diagnosis, staging, and consequently management of patients with prostate cancer (PCa). Although with less striking results, novel radiological modalities have also been proposed for bladder cancer (BCa) in recent years. Micro-ultrasound (MUS) is an imaging examination characterized by high real-time spatial resolution, recently introduced in the urological field. This article aimed to describe the current evidence regarding the application of MUS for the diagnosis and staging of PCa and BCa. *Materials and Methods*: We designed a narrative review. A comprehensive search in the MEDLINE, Scopus, and Cochrane Library databases was performed. Articles in English-language and published until July 2022 were deemed eligible. Retrospective and prospective primary clinical studies, as well as meta-analyses, were included. *Results*: MUS-guided prostate biopsy showed high sensitivity (0.91, 95% CI, 0.79–0.97) in the diagnosis of clinically significant PCa (csPCa). It was associated with a higher detection rate of csPCa than a systematic biopsy (1.18, 95% CI 0.83–1.68). No significant difference was found between MUS and mpMRI-guided biopsy in the total detection of PCa (*p* = 0.89) and in the detection of Grade Groups ≥ 2 (*p* = 0.92). The use of MUS to distinguish between non-muscle-invasive and muscle-invasive BCa was described, highlighting an up-staging with MUS only in a minority of cases (28.6%). *Conclusions*: Promising findings have emerged regarding the feasibility and accuracy of MUS in the diagnosis and staging of PCa and BCa. However, the available evidence is limited and should be considered preliminary.

## 1. Introduction

Great goals have been achieved in recent decades regarding the early diagnosis and staging of urological tumors, in particular of prostate (PCa) and bladder cancer (BCa) [1,2].

Current European Association of Urology (EAU) Guidelines recommend performing a multiparametric magnetic resonance imaging (mpMRI) before prostate biopsy in case of suspicious digital rectal examination (DRE) and/or elevated prostate-specific antigen (PSA) levels [3,4]. In addition to indicating the risk of a clinically significant PCa (csPCa) through the Prostate Imaging Reporting and Data System (PI-RADS) score in the diagnostic phase, mpMRI is the reference radiological modality for local staging [3,5]. Computed tomography (CT) and bone scan (BS) remain the imaging techniques indicated for the staging of distant metastases [3]. Transrectal ultrasound (TRUS) is not reliable in detecting PCa, and the diagnostic performance of additional biopsies performed on hypoechoic or color/power Doppler-positive lesions is controversial. Furthermore, TRUS seems no more accurate than DRE at predicting organ-confined PCa [6]. However, real-time ultrasound (US) guidance remains critical for performing both systematic and fusion prostate biopsies [7,8,9]. As part of the new imaging exams, prostate-specific membrane antigen positron emission tomography/CT (PSMA PET/CT) has gained great popularity in recent years. It is currently recommended in selected patients with biochemical recurrence (BCR) after curative treatment for PCa [3]. Several articles found a higher accuracy of PSMA PET/CT, compared with the common primary staging modalities (CT and BS) [10], and even sufficient accuracy for primary tumor detection; nevertheless, the possible benefits and harms resulting from these applications are under discussion [11]. Other radiological techniques for the diagnosis and staging of PCa are recently emerging, such as multiparametric US (mpUS), whole-body MRI (WB-MRI), and PSMA PET/MRI; however, they are still supported by limited evidence [12]. Several other imaging modalities were proposed to improve the management of patients with urological tumors (e.g., Near-Infrared Fluorescence), but very limited data are currently available in the PCa setting [13,14,15].

On the other hand, regarding the diagnosis of BCa, the first-level imaging technique in the case of hematuria is the US of the urinary tract. It can allow the detection of papillary BCa; however, US cannot reveal small or flat bladder tumors, and it can sometimes lead to false positives [16]. For these reasons, according to the current EAU Guidelines, the pivotal examination for the diagnosis of BCa remains cystoscopy [3,17]. Urine cytology and CT urography should be reserved for selected patients in whom a tumor of the urinary tract is suspected [18]. The histological diagnosis and local staging (T) of BCa are based on the transurethral resection of the bladder (TURB) [16]. Distant staging with CT is only recommended for those patients with muscle-invasive BCa (MIBC) due to its metastatic potential [19]. mpMRI may be useful to differentiate non-muscle-invasive BCa (NMIBC) (<T2) and MIBC (≥T2), using the Vesical Imaging Reporting and Data System (VI-RADS) score; however, the available evidence is still rather limited, and there is no specific recommendation in this regard [2].

Several limitations affect most of the diagnostic and staging techniques currently adopted for PCa and BCa. Limited accuracy, poor spatial resolution, the emission of ionizing radiation, the need for contrast agents, long duration, high costs, poor patient comfort, inadequate territorial diffusion, and specific contraindications variably impact the various radiological modalities available. Research in recent years has pushed toward the identification of new imaging examinations that could overcome these issues. Several studies on the diagnostic effectiveness of conventional brightness-mode (B-mode) US showed a poor efficacy of this imaging technique in PCa diagnosis, due to similar backscatter signals of malignant and healthy prostate parenchyma [20]. Micro-ultrasound (MUS) is based on B-mode images, but it involves the use of transducers operating at high frequency (up to 29 MHz), resulting in high real-time spatial resolution (down to 70 µm), which provides a significant improvement in the visualization of tissue details and planes, compared with conventional US (300% higher resolution). It is performed in a lithotomy position, transrectally in men (for PCa and BCa) and transvaginally in women (for BCa) [21]. The first urological studies on MUS date back to about ten years ago [22], and since then, several papers have been published. To date, promising but rather limited findings are available on the topic.

The aim of this study was to describe the current evidence regarding the application of MUS for the diagnosis and staging of PCa and BCa, highlighting the advantages and disadvantages of the technique.

## 2. Materials and Methods

We designed a narrative review of the studies investigating the role of MUS in the diagnostic and staging evaluation of PCa or BCa. A comprehensive search in the MEDLINE, Scopus, and Cochrane Library databases was performed. Different combinations of the following keywords were used according to a free-text protocol: “Micro-ultrasound”, “High resolution”, “Bladder”, “Prostate”, “Cancer”, “Tumor”, “Diagnosis”, “Staging”, and “Imaging”. Articles in English-language, published until July 2022, with no chronological restriction were deemed eligible. Retrospective and prospective primary clinical studies, both comparative and non-comparative, were included. Meta-analyses were also included. Conference abstracts, case reports, small case series (≤10 cases), editorials, narrative reviews, and animal studies were excluded. Studies not evaluating PCa or BCa in the setting of primary diagnosis or staging were excluded. The reference lists of the included papers were used to search other relevant articles. The selection of articles and data to be extracted was based on the authors’ expert opinions. According to the predefined study design, the results were qualitatively described, as reported in the primary studies, without quantitative synthesis.

## 3. Results

### 3.1. Potential Applications of MUS in PCa

This first study on MUS for the diagnosis of PCa was published by Pavlovich et al. in 2014.

**Figure 1:**
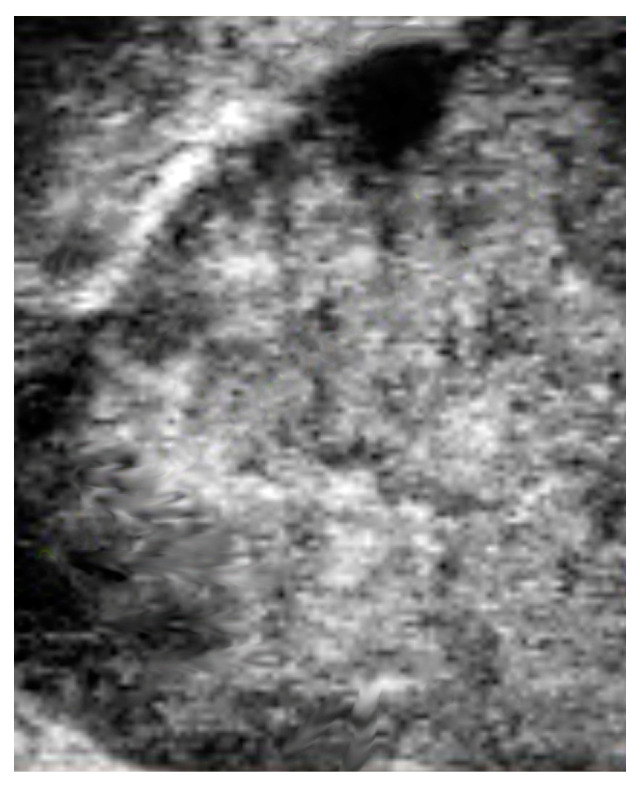
MUS of prostate: hypoechoic lesion and irregular prostate border.

The authors showed that this novel imaging modality had higher sensitivity (65.2% vs. 37.7%) and specificity (71.6% vs. 65.4%) than conventional US, paving the way for further research [22]. Another pivotal paper on the topic was written by Ghai et al. in 2016. In this article, the authors proposed Prostate Risk Identification using MUS (PRI-MUS), a 5-point grading system to stratify the risk of malignant prostatic lesions according to MUS images. This risk score was based on the ability of MUS to identify the histological changes typically associated with PCa due to its high spatial resolution (Figure 1). It is important to underline that the authors found that the PRI-MUS score was significantly increased by increasing the Gleason sum (severity of cancer) and the fraction of malignant tissue in the biopsy core (quantity of cancer) [23].

Several studies investigated the value of MUS as a guide to prostate biopsy to detect csPCa, comparing it with conventional US and mpMRI. In particular, the comparison with mpMRI was favored by the desire to overcome the limits and contraindications of this imaging modality.

In 2019, Zhang et al. assessed the accuracy of MUS-guided prostate biopsy in the diagnosis of csPCa with a meta-analysis including 7 studies and 769 patients. The authors found a pooled sensitivity of 0.91 (95% confidence interval (CI) 0.79–0.97) and a pooled specificity of 0.49 (95% CI 0.30–0.69), underlining the discussion that the detection ability of MUS was strong, but the possibility of misdiagnosis was high [24]. Pavlovich et al. published a prospective randomized controlled trial (RCT) in 2021, where 1676 men were randomized 1:1 to MUS vs. conventional US-guided prostate biopsy. The detection of csPCa with MUS was not superior in the overall population (34.6% vs. 36.6%; *p* = 0.21), increasing by 7% after standardized training [21]. In 2022, Dariane et al. compared MUS-guided with systematic prostate biopsy through a meta-analysis including 15 studies and 2967 patients. MUS-guided biopsies detected more csPCa (196 vs. 169 cases, detection ratio (DR): 1.18, 95% CI 0.83–1.68, I^2^ = 69%), and fewer non-csPCa, (62 vs. 115 cases, detection ratio 0.55, 95% CI 0.41–0.73, I^2^ = 0%) than systematic biopsies [25]. In 2019, Lughezzani et al. compared the diagnostic accuracy of MUS-guided prostate biopsy and MRI/US fusion-guided biopsy (reference test) in detecting csPCa in a prospectively collected cohort of 104 subjects with at least one PI-RADS ≥ 3 lesion. Sensitivity and negative predictive values (NPV) were 94% and 90%, respectively. On the other hand, a specificity value of 28% and a positive predictive value (PPV) of 40% were reported [26]. Klotz et al., in 2020, compared mpMRI and MUS for the detection of PCa in a multicenter prospective study on 1040 patients. Biopsies were taken from both mpMRI targets (PI-RADS ≥ 3) and MUS targets (PRI-MUS ≥ 3). The non-inferiority of MUS vs. mpMRI in the detection of csPCa was found for sensitivity (94% vs. 90%, *p* < 0.001), specificity (22% vs. 22%, *p* < 0.001), NPV (44% vs. 43%, *p* < 0.001), and PPV (85% vs. 77%, *p* < 0.001) [27]. In 2021, Sountoulides et al. published the first meta-analysis comparing the detection rate of MUS vs. mpMRI-guided prostate biopsy. The authors evaluated 18 articles involving 1125 men demonstrating similar detection rates between the techniques across all the PCa grades. The overall detection rate for the PCa and Grade Groups (GGs) ≥ 2 were 0.99 (95%, CI 0.89–1.11, I^2^ = 0%) and 1.05 (95%, CI 0.93–1.19, I^2^ = 0%), respectively [28]. These results were confirmed by You et al. in 2022 with a meta-analysis of 11 studies involving 1081 men. No significant difference was described between MUS and mpMRI-guided biopsy in the total detection of PCa (odds ratio (OR): 1.01, 95% CI: 0.85–1.21, *p* = 0.89) and in the detection of GGs ≥ 2 (OR: 1.01, 95% Cl: 0.83–1.22, *p* = 0.92) [29].

In 2020, Claros et al. compared the detection rates of MRI/MUS cognitive-guided biopsies and MRI/US fusion-guided biopsies in 269 patients. The MRI/MUS group (*n* = 47) was associated with a higher detection rate of csPCa than the MRI/US group (*n* = 222) (38% vs. 23% csPCa; *p* = 0.02). Despite these data considering PCa detection, regardless of the Gleason score or by random plus target biopsies, no significant difference was found between the groups [30]. In 2021, Avolio et al. retrospectively evaluated 111 patients scheduled for a prostate biopsy with at least one PI-RADS 3 lesion at mpMRI to verify if MUS could help substratify the risk of csPCa. Considering csPCa, MUS showed high sensitivity and NPV (100%). Overall, 27% of the patients could have safely avoided a prostate biopsy using MUS to stratify risk [31]. Preliminary evidence also highlighted a potential role in PCa staging for MUS. In 2022, Fasulo et al. published a prospective study on 140 patients with biopsy-proven PCa scheduled for robot-assisted radical prostatectomy, evaluating the accuracy of MUS in predicting extraprostatic extension (EPE). The authors found sensitivity, specificity, NPV, and PPV values of 72.1%, 88%, 80.5%, and 83.0%, respectively. The most important predictor of EPE was the presence of a visible capsular breach at MUS [32]. Overall, several authors indicate MUS as a less expensive and accessible alternative to mpMRI [33]. It is important to underline that every US technique, including MUS, is strictly operator-dependent. Only a few studies specify that operators had adequate training with MUS [23,27,31]; therefore, this could strongly influence the results found. The studies included in this review and their main results are summarized in Table 1.

### 3.2. Potential Applications of MUS in BCa

The literature on MUS in BCa patients is still very limited. Differentiation between MIBC and NMIBC is essential, both because it changes the prognosis and because it strongly influences patient management [34]. This distinction is normally entrusted to TURB, an invasive endoscopic procedure that generally involves hospitalization, the need for an operating room, and a certain complication rate. Furthermore, in some patients, a second TURB may be indicated for incomplete resection, the absence of muscle in the specimen, or T1 tumors [35]. In order to avoid the risks related to TURB, especially in those patients with severe comorbidities or taking anticoagulants, the development of a non-invasive procedure for local staging of BCa would be desirable. CT is generally used for BCa staging and provides few details about tumor invasion into the muscularis propria [36]. In this context, the mpMRI of the bladder has recently been proposed. It provides a fairly accurate estimate of the risk of muscle invasion using the VI-RADS score [2]. However, the local staging of BCa with mpMRI is not yet recommended or widely used, first of all, due to the limited evidence available, and secondly, due to some relevant limitations such as high costs, the limited availability of trained radiologists, the possible artifacts caused by previous TURB or intravesical therapy, the risk of over-staging caused by tumor-associated fibrosis, and the need for contrast agents with a risk of adverse effects (especially in patients with renal diseases or allergic subjects) [37]. In 2020, Saita et al. published an observational prospective study evaluating the MUS in 23 patients (12 males and 11 females) with primary BCa for the discrimination of NMIBC and MIBC [38]. MUS findings were correlated with those of histological analysis after TURB. The pathologists confirmed all the 14 NMIBCs, while 2/7 MIBCs (28.6%) were down-staged to NMIBCs at assessment. The examination was impossible in two men with large prostates (longitudinal diameter > 5 cm) because it limited the bladder window. All tumors ≥ 5 mm were clearly visualized. The technique provided only the longitudinal sections of the bladder; therefore, the evaluation of the lesions localized in the lateral walls could be suboptimal. No differentiation was performed between Ta and T1 tumors. No carcinoma in situ (CIS) was studied, and only one flat tumor (T1) was included in the analysis. Although this paper shows encouraging results, it has important limitations such as the small sample size and remains to date the only clinical study available in the literature on the topic.

It is essential to underline that a significant intrinsic limitation of all radiological techniques is the impossibility of obtaining a histological sample. Therefore, TURB remains the fundamental procedure for histological diagnosis and the first treatment of bladder lesions, and as it is practiced for these purposes, it is obvious that it will continue to be used for local staging (T parameter). Consequently, imaging could primarily play a complementary role to the TURB to reduce the need for an unnecessary second TURB, or conversely, to direct toward a second TURB when this seems not indicated.

The advantages and disadvantages of MUS in patients with PCa and BCa were summarized in Table 2.

### 3.3. Strengths, Limitations, and Future Perspectives

To the best of our knowledge, this is the most up-to-date review on the topic. We described the most current evidence on MUS, highlighting its potential, benefits, and limitations in patients with PCa and BCa. However, this paper should be read considering several limitations. The main weakness is the narrative design and the consequent absence of quantitative synthesis of the data (meta-analysis). However, the paucity, heterogeneity, and low-to-intermedium quality of the available data may have made the data synthesis unreliable. This limitation also impacted the quality of the meta-analyses published on the topic and included in our article. Due to these issues, it was not possible to provide specific recommendations on MUS.

Future research should produce well-designed RCTs, in which MUS is compared with reference imaging techniques and histological analysis. This appears even more important in the context of BCa, where evidence is still very limited at present. Specific studies to investigate the possible overdiagnosis and overtreatment of PCa associated with MUS should be designed. Further efforts should be made to standardize the technique and interpretation of MUS images. The learning curve associated with MUS should be evaluated with appropriate studies. Moreover, the greater territorial availability of the technology would be desirable to increase the clinical experience and confidence in MUS. The ultimate goal of research should be to produce sufficient evidence to allow scientific societies to provide clear recommendations.

## 4. Conclusions

MUS is a novel radiological technique that has been proposed for the diagnosis and staging of PCa and BCa. This modality seems to be able to overcome, at least partially, the limitations of the imaging commonly used, and promising results have emerged regarding its feasibility and accuracy. However, MUS is not without its disadvantages, and the supporting clinical evidence is still limited, especially in the context of BCa. For this reason, the encouraging data available should be considered preliminary. Further well-designed studies are needed to improve the evidence on the topic, promote the dissemination of the technique, and provide clear recommendations.

## Figures and Tables

**Table 1:** Articles on MUS in PCa settings included in the review.

First Author and Publication Year	Objectives	Main Findings
C. P. Pavlovich et al., 2014 [22]	Proposal of MUS for diagnosis of PCa	MUS had higher sensitivity (65.2% vs. 37.7%) and specificity (71.6% vs. 65.4%) than conventional US in the diagnosis of PCa.
S. Ghai et al., 2016 [23]	Proposal of PRI-MUS	10.1% increase (95% CI 9.3–10.8) in the probability of csPCa.
M. Zhang et al., 2019 [24]	Assessment of the accuracy of MUS-guided prostate biopsy in the diagnosis of csPCa (meta-analysis)	MUS pooled sensitivity, specificity, DOR, and an area under the SROC of 0.91, 0.49, 10, and 0.82, respectively. MUS has a superior ability to diagnose csPCa.
G. Lughezzani et al., 2019 [26]	Evaluation of diagnostic accuracy of MUS-guided prostate biopsy and MRI/US fusion-guided biopsy in detecting csPCa	PCa was diagnosed in 56 patients (54%) and csPCA in 35 (34%). MUS sensitivity for csPCA detection was 94%, with 33/35 csPCA cases correctly identified. The negative predictive value was 90%, while the positive predictive value was 40% and the specificity was 28%. Of the 61 targeted zones concordant between MUS and mpMRI, 24 were csPCA. Discordant targeted lesions led to csPCA discovery by MUS in three cases and mpMRI in four cases. Both techniques missed one case for which csPCA was diagnosed by systematic biopsies only.
L. Klotz et al., 2020 [27]	Comparison of mpMRI and MUS for the detection of PCa	MUS and mpMRI sensitivity was 94% vs. 90%, respectively (*p* = 0.03), and NPV was 85% vs. 77%, respectively. Specificities of MUS and MRI were both 22%, with similar PPV (44% vs. 43%).
O. R. Claros et al., 2020 [30]	Comparison of the DR of MRI/MUS cognitive-guided biopsies and MRI/US fusion-guided biopsies	In targeted biopsies, MUS biopsy cases presented higher detection of csPCa than the robotic ultrasound magnetic resonance imaging fusion biopsy group (38% vs. 23%, *p* = 0.02).
C. P. Pavlovich et al., 2021 [21]	Comparison of MUS vs. conventional US-guided prostate biopsy in the detection of csPCa	Detection of csPCa with MUS was not superior in the overall population (34.6% vs. 36.6%; *p* = 0.21).
P. Sountoulides et al., 2021 [28]	Comparison of DR of MUS vs. mpMRI-guided prostate biopsy (meta-analysis)	The pooled DR for International Society of Urological Pathology Grade Group ≥2 prostate cancer was 1.05 (95% CI 0.93–1.19, I^2^ = 0%), 1.25 (95% CI 0.95–1.64, I^2^ = 0%) for Grade Group ≥3 and 0.94 (95% CI 0.73–1.22, I^2^ = 0%) for clinically insignificant (Grade Group 1) prostate cancer. The overall DR for PCa was 0.99 (95% CI 0.89–1.11, I^2^ = 0%).
C. You et al., 2021 [29]	Comparison of DR of MUS vs. mpMRI-guided prostate biopsy (meta-analysis)	The meta-analysis showed that no significant difference was found between MUS and mpMRI-TB in the total detection of PCa (odds ratio (OR): 1.01, 95% confidence interval (CI): 0.85–1.21, *p* = 0.89), of grading groups (GGs) = 1 (OR: 0.92, 95% CI: 0.68–1.25, *p* = 0.59), of GGs ≥2 (OR:1.01, 95% CI: 0.83–1.22, *p* = 0.92), and of GGs ≥3 (OR: 1.31, 95% CI: 0.95–1.81, *p* = 0.10).
P. P. Avolio et al., 2021 [31]	Evaluation of the ability of MUS to substratify the risk of csPCa in patients with PI-RADS 3 lesions on mpMRI	MUS showed a high sensitivity and negative predictive value (100%), while its specificity and positive predictive value were 33.7% and 27.2%, respectively. Among patients without lesions at MUS, 25 (83.3%) did not harbor PCa, while 5 (16.7%) patients were diagnosed with a Gleason score of 6 PCa, with no patients harboring csPCa. Using MUS, the csPCa detection would have remained at 100% while reducing the detection of insignificant PCa to a 23.8% extent (*n* = 5).
C. Dariane et al., 2022 [25]	MUS-guided vs. systematic prostate biopsy in PCa diagnosis (meta-analysis)	Overall, PCa was detected in 56–71% of men, with 31.3–49% having csPCa and 17–25.4% having non-csPCa. Regarding csPCa, MUS-guided biopsies identified 196 and SB 169 cases (DR: 1.18, 95% CI 0.83–1.68, I2 = 69%), favoring MUS-guided biopsies; regarding non-csPCa, MUS-guided biopsies identified 62 and SB 115 cases (DR: 0.55, 95% CI 0.41–0.73, I2 = 0%), also favoring MUS-guided biopsies by decreasing unnecessary diagnosis.
V. Fasulo et al., 2022 [32]	Evaluation of the accuracy of MUS in predicting EPE	The presence of visible extracapsular extension at MUS predicted EPE with a sensitivity of 72.1% and a specificity of 88%, an NPV of 80.5%, and a PPV of 83.0%, with an AUC of 80.4%.

MUS: micro-ultrasound; PCa: prostate cancer; csPCa: clinically significant prostate cancer; mpMRI: multiparametric MRI; mpMRI-TB: multiparametric magnetic resonance imaging-targeted biopsy; EPE: extraprostatic extension; NPV: negative predictive value; PPV: positive predictive value; CI: confidence interval; DOR: diagnostic odds ratio; DR: detection rate; AUC: area under the curve; PRI-MUS: Prostate Risk Identification using MUS; PI-RADS: Prostate Imaging Reporting and Data System.

**Table 2:** MUS in PCa and BCa: pros vs. cons.

Pros	Cons
Non-invasive Absence of ionizing radiation No contrast agents Short duration * Practicable directly from the urologist (in addition to the radiologist) High spatial resolution Real-time visualization Cost-effective * Readily accessible Encouraging preliminary data **	Limited evidence, especially for BCa ** Need for specific technology/devices and training: current limited availability Need for further standardization of technique and interpretation Operator dependent technique Impossibility of histological sampling (without other complementary procedures) Lithotomic position: prolonged positioning time, need for an adequate medical bed, contraindication in case of hip problems Endocavitary approach: reduced patient comfort, contraindication in case of anal or vaginal stricture Need for rectal (e.g., enema) or bladder (e.g., filling with saline) preparation Reduced wave penetration depth (6 cm) Specific limitations referred to PCa: possible US signal loss in the deeper anterior zone in case of large prostate, low specificity (high possibility of misdiagnosis) Specific limitations referred to BCa: limited bladder window in case of large prostate, suboptimal evaluation of lesions in the lateral bladder wall, possible difficulty in identifying flat lesions

MUS: micro-ultrasound; PCa: prostate cancer; BCa: bladder cancer; US: ultrasound. * Hypothesized feature. Need for specific studies. ** Refer to the main text.

## Data Availability

Not applicable.
